# Accuracy of the Injection-Based Occlusion Tool Utilizing Saline and Glucose Solution in Cryoballoon Ablation Guided by a Novel Dielectric Imaging System

**DOI:** 10.3390/jcdd10100427

**Published:** 2023-10-17

**Authors:** Binfeng Mo, Jiali Yuan, Xiaoming Lian, Xingxing Cai, Qunshan Wang, Yigang Li

**Affiliations:** Department of Cardiology, Xinhua Hospital Affiliated to Shanghai Jiao Tong University School of Medicine, #1665 Kong Jiang Road, Shanghai 200092, China

**Keywords:** atrial fibrillation, cryoballoon ablation, new dielectric imaging system, occlusion tool, pulmonary vein isolation

## Abstract

**Introduction:** The aim of this study is to assess the accuracy of the injection-based occlusion (IBO) tool utilizing saline and glucose solution in verifying pulmonary vein (PV) occlusion during cryoballoon ablation guided by a novel dielectric system (KODEX–EPD system). **Methods:** In this retrospective study, we enrolled 34 consecutive patients with paroxysmal atrial fibrillation (AF) who underwent their initial cryoballoon ablation procedure guided by the KODEX-EPD system. PV occlusion was firstly assessed by the IBO tool utilizing saline or glucose solution and then verified by direct contrast angiography. Patients were divided into two groups according to the fluid used in the IBO tool: the Saline Group and the Glucose Group. **Results:** The overall procedure time and fluoroscopy time were comparable between the Saline Group and the Glucose Group (113.7 ± 18.3 vs. 108.4 ± 15.9 min; *p* = 0.375 and 10.1 ± 3.7 vs. 9.3 ± 3.5 min; *p* = 0.559). The IBO tool was utilized a total of 138 times in the Saline Group and 135 times in the Glucose Group. When assessing PV occlusion, the IBO tool using saline demonstrated a sensitivity of 92.6% and a specificity of 95.2% compared to angiography. Similarly, the IBO tool utilizing glucose solution showed a sensitivity of 93.2% and a specificity of 96.1%. **Conclusions:** The IBO tool utilizing non-contrast fluid, saline and glucose solution, demonstrates a high level of sensitivity and specificity in accurately predicting PV occlusion during cryoablation procedures. Both the saline and glucose solutions used in the IBO tool show promising results in effectively assessing PV occlusion.

## 1. Introduction

Pulmonary vein (PV) isolation is the cornerstone of catheter ablation for atrial fibrillation (AF) [1]. Cryoballoon-based PV isolation has been shown equally safe and effective as compared to radiofrequency ablation in the randomized study of FIRE AND ICE [2]. Recent evidences support cryoballoon ablation as a first-line treatment for paroxysmal AF [3,4]. However, the traditional method of assessing PV occlusion during cryoballoon ablation, using angiography via the tip of the cryoballoon, involves higher radiation exposure and dye usage compared to radiofrequency ablation.

To address this, the KODEX–EPD (EPD Solutions, Philips, the Netherlands) imaging system has been developed. It is a novel 3D mapping system utilizing wide-band dielectric technology. This open platform is compatible with validated electrophysiology catheters and has the potential to generate computed tomography–like images [5]. Moreover, it incorporates two occlusion assessment software tools: the baseline occlusion tool and the injection-based occlusion (IBO) tool, which enable non-fluoroscopic assessment of PV occlusion.

The initial results of the IBO tool using dye have shown a sensitivity of 91% and a specificity of 96% in assessing complete PV occlusion, surpassing the accuracy of the baseline tool [6]. However, the use of contrast medium remains a concern. The IBO tool detects residual PV leaks by sensing dielectric changes through the electrodes of the circular mapping catheter after the injection of a solution with different conductivity than blood. Considering the significant differences in conductivity between saline or glucose solution and blood, it is hypothesized that the IBO tool utilizing either saline or glucose solution can provide accurate assessment of PV occlusion while reducing the need for contrast dye.

Therefore, the objective of this study was to evaluate the accuracy of the IBO tool utilizing saline and glucose solutions in verifying PV occlusion during cryoballoon ablation guided by the novel dielectric system.

## 2. Methods

### 2.1. Study Population

This retrospective study enrolled consecutive patients with symptomatic paroxysmal AF who underwent their initial cryoballoon ablation procedure between September 2021 and August 2022. The study included patients aged 18 years and above who had drug-resistant paroxysmal atrial fibrillation (AF) and underwent cryoballoon-based pulmonary vein (PV) isolation guided by the KODEX-EPD system. Patients with persistent AF, previous AF ablation, contrast media allergies, hyperthyroidism, or renal dysfunction were excluded from the study.

The accuracy of PV occlusion was assessed using the injection-based occlusion (IBO) tool with either 0.9% saline (Saline Group) or 10% glucose solution (Glucose Group). The evaluation was based on angiograms obtained by injecting dye into each PV. Radiation exposure and dye usage were also documented.

The retrospective study was approved by the Ethics Committee of Xinhua Hospital affiliated to Shanghai Jiao Tong University School of Medicine and complies with the Declaration of Helsinki. Written informed consent was obtained from all participating patients.

### 2.2. Cryoballoon Ablation Procedure

The procedure was conducted with the patient under local anesthesia and conscious sedation. To begin, a decapolar catheter was inserted into the coronary sinus through the right femoral vein. Following a successful transseptal puncture, the transseptal sheath was exchanged with a steerable 15 Fr sheath (FlexCath Advance, Medtronic, Minneapolis, MN, USA) over a stiff guidewire. A second-generation 28 mm cryoballoon catheter (Arctic Front Advance, Medtronic, Minneapolis, MN, USA) was then advanced through the steerable sheath into the left atrium, with a 20-mm small-diameter circular mapping catheter (Achieve, Medtronic, Minneapolis, MN, USA) inserted into the central lumen of the cryoballoon to serve as a guidewire. Throughout the procedure, the activated clotting time was maintained between 250 and 350 s using unfractionated heparin.

**Figure 1:**
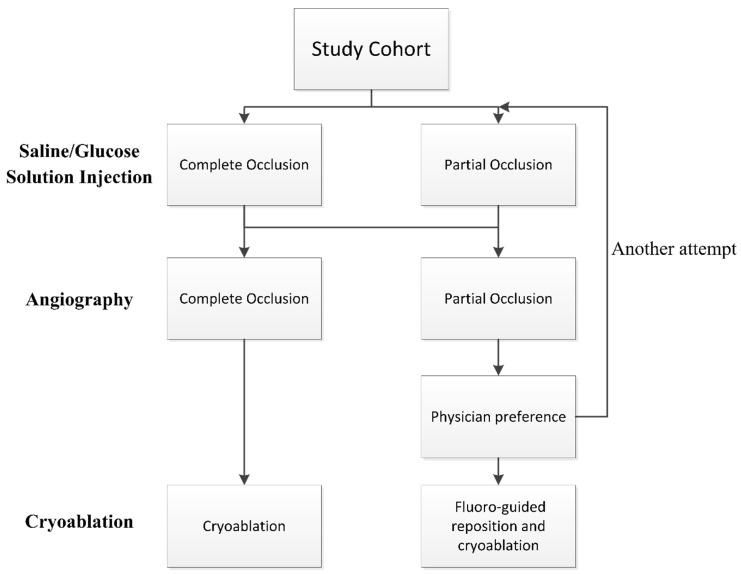
Flowchart of the procedure. Pulmonary vein occlusion is firstly assessed by injection-based occlusion tool utilizing saline or glucose solution and then verified by direct contrast angiography.

The positioning of the balloon was visualized using fluoroscopy and the KODEX-EPD system. Before each PV cryoablation, the circular catheter was positioned at the ostium of the PV. The inflated cryoballoon was then placed at the PV ostium to occlude it. The occlusion of the PV was initially assessed using the IBO tool with saline or glucose solution, and subsequently confirmed through direct contrast angiography. Complete occlusion was defined as total contrast retention without any backflow to the atrium during selective PV venography. If angiography indicated complete occlusion, cryoablation was applied. In case of partial occlusion, the occlusion tool was attempted again or conventional fluoro-guided cryoablation was performed based on the physician’s preference. See Figure 1 for details of the flowchart.

The standard sequence of ablation started with the left superior pulmonary vein (LSPV) as the first target, followed by the left inferior (LIPV), right superior (RSPV), and right inferior (RIPV) veins. PV potentials were tried to recorded before and after complete PV isolation using the circular catheter. A time-to-isolation (TTI) guided protocol was employed in all cases, with a standard freezing time of 180 s [7]. An additional application of 120 s was performed if a TTI of 60 s or longer was observed, or if no TTI was documented after acute electrical disconnection. During freezing at the right-sided PVs, the right phrenic nerve was paced (20 mA at 2.0 msec pulse width at a cycle length of 1500 msec) from the superior caval or subclavian vein. Phrenic capture was monitored through tactile feedback, and ablation was immediately stopped upon the cessation or weakening of diaphragmatic contraction.

### 2.3. The KODEX-EPD System and Injection-Based Occlusion Tool

**Figure 2:**
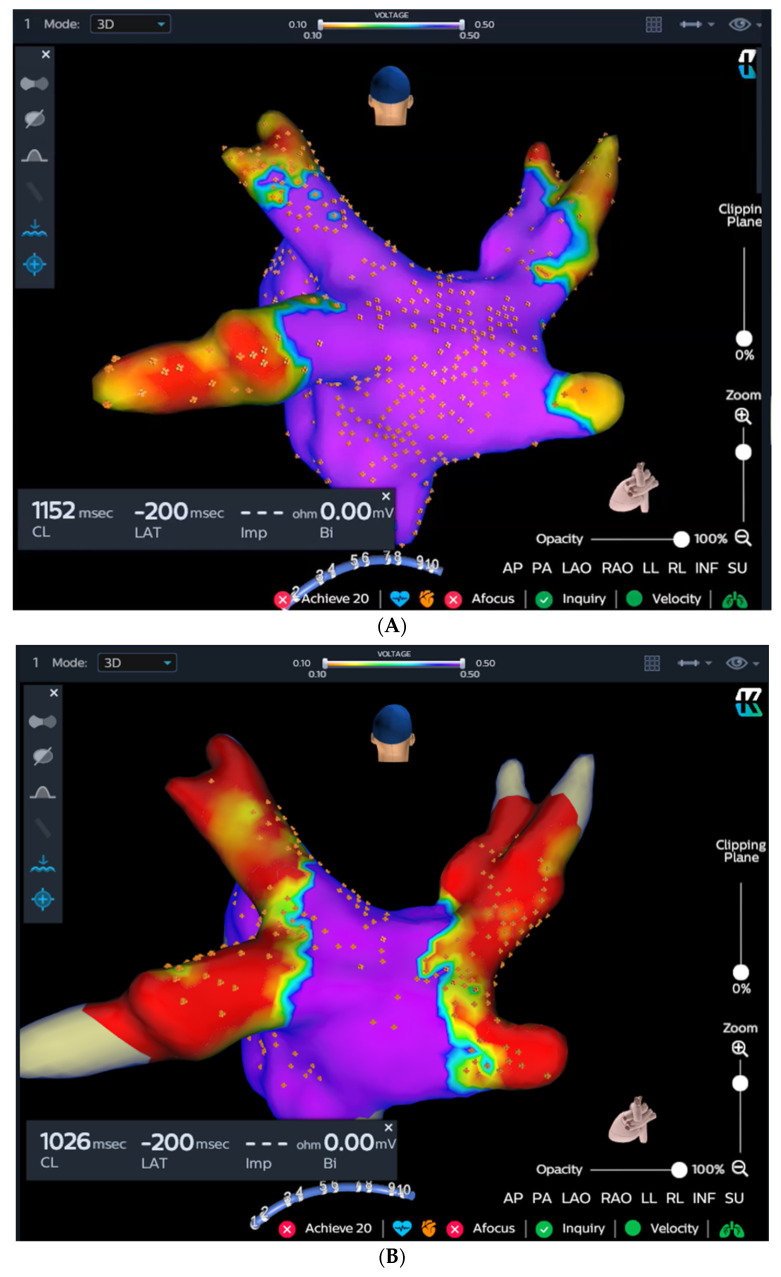

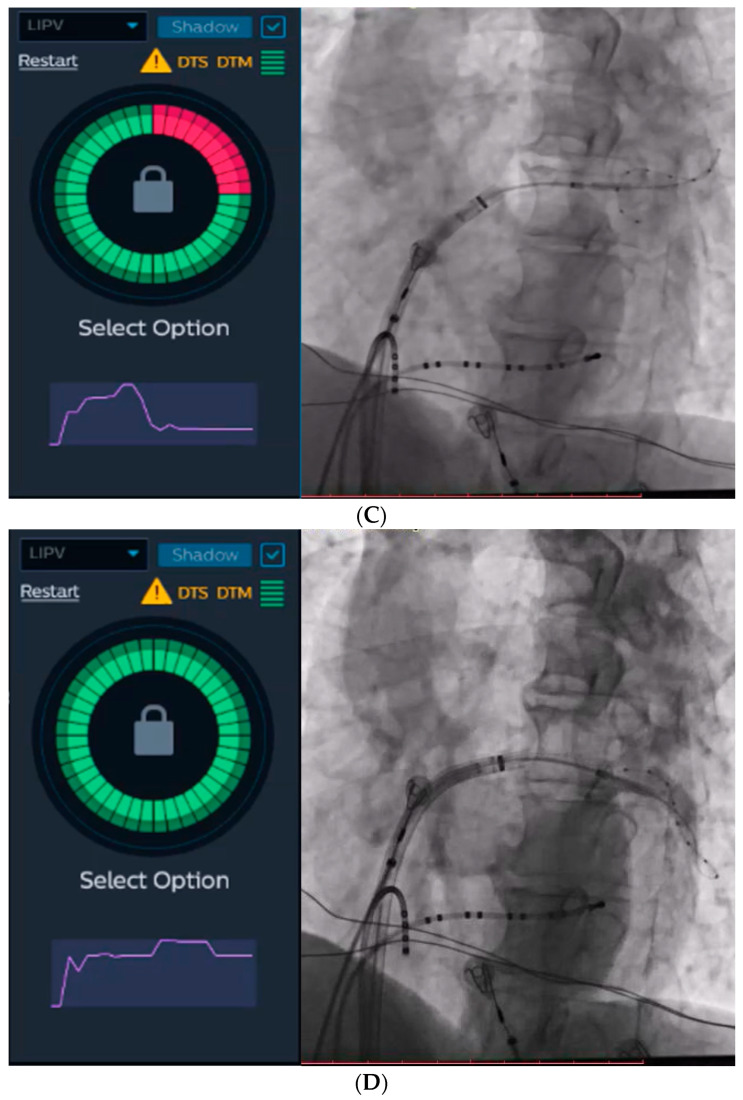
The KODEX-EPD system and injection-based occlusion tool. Left atrial dielectrical anatomy and voltage mapping prior to cryoablation (**A**) and at the end of the procedure (**B**). (**C**) Since the graph returns to baseline after injection, the tool suggests a partial occlusion (**left**), which is verified by contrast angiography (**right**). (**D**) Since the graph forms a plateau after the injection, the tool suggests a complete occlusion (**left**), which is verified by contrast angiography (**right**).

The KODEX-EPD system creates high-resolution images of cardiac anatomy by exploiting the distinct dielectric properties of biological tissue [5,8]. In this study, the KODEX-EPD system’s R1.4.8 software version was employed. Utilizing a circular catheter, the left atrial anatomy was obtained automatically by the system. Voltage mapping was conducted before cryoablation (Figure 2A) and upon completion of the procedure (Figure 2B).

The KODEX-EPD system incorporates an IBO tool that utilizes dye to evaluate PV occlusion. For the purpose of this study, we employed saline and glucose solution for injection. During this particular step, a rapid injection of 10–12 mL of either saline or glucose solution was administered into the PV. Simultaneously, a dissipation waveform graph was displayed, depicting the average change in local dielectric properties caused by the injected solution. This graph was captured by the circular catheter. If the graph returns to its baseline after the injection, the IBO tool suggests a partial occlusion (Figure 2C). Conversely, if the graph reaches a plateau, it indicates a complete occlusion with stasis in the PV (Figure 2D). The system provides a color-coded qualitative indication of the occlusion state, with green representing complete occlusion and red representing partial occlusion.

### 2.4. Follow-Up

Post-discharge, follow-up visits were scheduled for patients at the 1, 3, 6, and 12-month marks after the procedure, with subsequent visits scheduled semi-annually thereafter. These visits could be conducted either in the office or via transtelephonic means. Arrhythmia monitoring during these visits involved 12-lead electrocardiography or 24-h Holter monitoring. Additionally, monitoring was conducted when patients experienced symptoms following the blanking period. Atrial arrhythmia recurrence was defined as documented atrial fibrillation, atrial tachycardia, or atrial flutter lasting more than 30 s. If no clinical or documented atrial fibrillation recurrences were observed after the blanking period, antiarrhythmic drug therapy was discontinued after 3 months. Anticoagulation therapy was continued for a minimum of 3 months and then determined based on individual CHA2DS2-VASc scores [1].

### 2.5. Statistical Analysis

Continuous variables were described as mean ± standard deviation (median [interquartile range] for non-normal data) and compared using Student’s t-test (Mann–Whitney U test if normality not satisfied). Categorical variables were presented as percentages and were analyzed using chi-square test or Fisher exact test where appropriate. A two-tailed probability value of <0.05 was considered statistically significant. All statistical analyses were conducted using SPSS version 22.0 (IBM Software Inc., Armonk, NY, USA).

## 3. Results

### 3.1. Baseline Characteristics

A total of 17 patients were consecutively enrolled in each group. The baseline clinical and demographic characteristics of the patients are summarized in Table 1. The mean age of the Saline Group was 63.6 ± 9.0, while the Glucose Group had a mean age of 63.1 ± 7.7 (*p* = 0.643). In terms of gender composition, women accounted for 41.2% in the Saline Group and 52.9% in the Glucose Group. The patients included in the study exhibited overall good health with limited comorbidities, except for hypertension. Both groups had similar low CHA2DS2-VAS scores, with values of 1.3 ± 1.0 in the Saline Group and 1.1 ± 1.1 in the Glucose Group (*p* = 0.646). The left atrial diameter measured 36.6 ± 3.0 mm in the Saline Group and 37.1 ± 3.0 mm in the Glucose Group (*p* = 0.612).

### 3.2. Ablation Procedure and Short-Term Outcome

Acute cryoballoon-based PV isolation was successful in all the patients. Among them, one patient in the Saline Group and two patients in the Glucose Group had a left common ostium. A single freeze was able to achieve PV isolation in 57 out of 67 PVs (85.1%) in the Saline Group and 54 out of 66 PVs (81.8%) in the Glucose Group. The overall procedure time was comparable between the Saline Group and the Glucose Group, with values of 113.7 ± 18.3 min and 108.4 ± 15.9 min, respectively (*p* = 0.375). Similarly, the fluoroscopy time was 10.1 ± 3.7 min in the Saline Group and 9.3 ± 3.5 min in the Glucose Group (*p* = 0.559). The amount of dye used during the procedure was 24.3 ± 7.8 mL in the Saline Group and 21.8 ± 6.2 mL in the Glucose Group. No major complications were observed, except for one patient in the Saline Group who experienced a mild groin hematoma, which resolved without the need for surgery.

At a mean follow-up of 8.2 ± 2.9 months in the Saline Group and 7.9 ± 2.7 months in the Glucose Group, the success rates after the blanking period were 82.4% and 88.2%, respectively.

### 3.3. Injection-Based Occlusion Tool Performance

In the Saline Group, a total of 138 evaluations using the IBO tool with saline were performed on 66 PVs. The performance of the IBO tool utilizing saline in assessing PV occlusion, compared with angiography, is presented in Table 2. The sensitivity [true positive/(true positive + false negative)] was in total 92.6%, and 100.0%, 91.7%, 100.0%, 72.7% for LSPV, LIPV, RSPV and RIPV, respectively. The specificity [true negative/(true negative + false positive)] was in total 95.2%, and 93.8%, 95.8%, 100.0%, 93.5% for LSPV, LIPV, RSPV and RIPV, respectively.

In the Glucose Group, a total number of 135 evaluations using the IBO tool with 10% glucose solution were analyzed in 64 PVs. The sensitivity was in total 93.2%, and 100.0%, 92.9%, 100.0%, 75.0% for LSPV, LIPV, RSPV and RIPV, respectively. The specificity was in total 96.1%, and 100.0%, 95.7%, 100.0%, 92.9% for LSPV, LIPV, RSPV and RIPV, respectively.

The positive and negative predictive values of the two groups were also reported in Table 2.

## 4. Discussions

The current study is the first to evaluate the accuracy of the IBO tool using non-contrast fluids, saline and glucose solution, in confirming PV occlusion during cryoballoon ablation. The findings demonstrated that both the IBO tool with saline and glucose solution exhibited high sensitivity and specificity in predicting PV occlusion. These results suggest that the IBO tool with non-contrast fluids has the potential to reduce or even eliminate the need for contrast injection during cryoablation procedures.

### 4.1. The KODEX-EPD System and Occlusion Tools

Cryoablation has been shown to be a safe and effective method for PV isolation, comparable to radiofrequency ablation. However, traditional fluoroscopic-guided cryoablation is associated with higher fluoroscopy exposure and dye use, although it offers advantages such as a shorter learning curve and time-saving benefits. To address these challenges, the KODEX-EPD imaging system, which utilizes dielectric sensing, has been introduced as a non-fluoroscopic imaging tool for atrial anatomy visualization, catheter navigation, and assessment of PV occlusion using two occlusion tools.

The baseline occlusion tool was initially introduced in version 1.4.6 of the KODEX-EPD imaging system. This tool involves recording baseline dielectric parameters using a circular catheter within the PVs and comparing them with dielectric parameters obtained when the inflated balloon is advanced against the PV ostium [9]. In a study of 28 patients, the baseline occlusion tool demonstrated a sensitivity of 91% and a specificity of 76% in assessing complete PV occlusion, which was verified with contrast medium injection [9]. Another study involving 82 patients reported a sensitivity of 80% and a specificity of 91% for the baseline occlusion tool [10]. A subsequent case-control study compared fluoroscopy and dye use in cryoballoon ablation between the KODEX-EPD imaging system guidance (utilizing the baseline occlusion tool) and traditional fluoroscopic guidance in a sample of 34 patients [11]. The study found significantly lower fluoroscopy time (8 min, interquartile range [IQR] 5–9 min vs. 11 min, IQR 9–12 min; *p* = 0.014) and reduced dye usage (35 mL, IQR 28–45 mL vs. 70 mL, IQR 57–83 mL; *p* < 0.001) in the KODEX-EPD group compared to the traditional fluoroscopic guidance group.

The IBO tool for the occlusion assessment was lately added in version 1.4.7. This tool confirms occlusion status by sensing the dielectric changes by the electrodes of the circular catheter after injection of solution whose conductivity is different from blood. However, it’s important to note that the algorithm of the IBO tool in versions 1.4.7 and 1.4.8 is specifically designed for contrast medium, which means that contrast injections are still required for its use.

The IBO tool is expected to provide higher accuracy compared to the baseline workflow, particularly in detecting smaller leaks. In an initial study of 50 patients, the IBO tool utilizing dye demonstrated a sensitivity of 91% and a high specificity of 96% in assessing complete PV occlusion [6]. In our study, the IBO tool utilizing saline showed a sensitivity of 92.6% and a specificity of 95.2%, while the injection of glucose solution demonstrated a sensitivity of 93.2% and a specificity of 96.1%. These results indicated that the injection of non-contrast solutions, such as saline and glucose solution, yielded accuracy comparable to dye injection when using the IBO tool of the KODEX-EPD system.

Saline and glucose solution have similar viscosity to water, making it possible to inject them in high volumes. They also allow better penetration of the PVs and can be quickly washed out. The potential benefits of utilizing saline and glucose solution may include

(a) reduction of the contrast-induced kidney injury;
(b) higher confidence in occlusion assessment and better sensitivity to small leaks;
(c) shorter assessment time.

### 4.2. Technical Thinking

An analysis of the performance of the IBO tool in different PVs revealed significant variability. Superior PVs demonstrated near-perfect sensitivity and specificity, suggesting that, in superior PVs, contrast angiography may be unnecessary after occlusion confirmation with the injection tool. However, the accuracy of the IBO tool was notably lower in inferior PVs, particularly in the RIPVs. This discrepancy can be attributed to the challenging anatomies of the RIPVs.

During ablation of the RIPV, the circular catheter is typically positioned deep inside the PV to provide better support. However, if the circular catheter is positioned too far from the balloon, it may fail to detect the dielectric changes caused by solution injection, resulting in false negatives. Additionally, the circular catheter may detect dielectric changes due to solution flow between PV branches, leading to false negatives as well. Consequently, the sensitivity of the RIPVs was considerably lower compared to other PVs.

It is worth noting that the current version of the KODEX-EPD system and the IBO tool do not offer significant value in terms of balloon positioning for the RIPVs. However, the future version of the KODEX-EPD system might incorporate cryoballoon visualization, which could aid in the positioning of the cryoballoon and potentially reduce radiation exposure and dye usage during the procedure. This advancement could enhance the overall accuracy and efficiency of cryoballoon ablation, particularly in challenging anatomical regions like the RIPVs.

## 5. Limitations

The pilot study described in the previous discussion has several limitations that should be acknowledged. Firstly, the study had a relatively small sample size, which may limit the generalizability of the findings. Additionally, the study was conducted retrospectively, which introduces potential biases and limitations inherent to retrospective designs.

Another limitation is that while the descending slope of the waveform graph after injection can provide some indication of the extent of leakage, the current version of the KODEX-EPD system does not offer a quantitative assessment of the leakage. This lack of quantitative assessment may impact the accuracy and precision of the occlusion evaluation.

Furthermore, it’s important to note that the data in the study was collected using version 1.4.8 of the KODEX-EPD system. However, the saline injection workflow is now supported in the KODEX version 1.5.1, which is expected to provide improved performance compared to the version used in this study. Therefore, the results and conclusions from the pilot study may not fully reflect the capabilities and accuracy of the latest version of the KODEX-EPD system.

Future studies with larger sample sizes and prospective designs should be conducted to validate the findings and assess the performance of the KODEX-EPD system.

## 6. Conclusions

The IBO tool utilizing saline and glucose solution demonstrates high sensitivity and specificity in predicting PV occlusion during cryoablation. The data presented in this paper emphasize its potential value, particularly in patients with renal insufficiency undergoing cryoballoon ablation.

## Figures and Tables

**Table 1:** Baseline characteristics.

	Saline Group N = 17	Glucose Group N = 17	*p* Value
Age, years	63.6 ± 9.0	63.1 ± 7.7	0.643
Female	7 (41.2%)	9 (52.9%)	0.492
BMI, kg/m^2^	23.3 ± 3.2	24.6 ± 2.8	0.220
AF history, months	13.3 ± 11.7	11.5 ± 9.1	0.626
Paroxysmal AF	100 (100.0%)	100 (100.0%)	-
Coronary artery disease	1 (5.9%)	2 (11.8%)	0.545
Hypertension	7 (41.2%)	6 (35.3%)	0.724
Heart failure	0 (0.0%)	0 (0.0%)	-
Diabetes mellitus	1 (5.9%)	2 (11.8%)	0.545
Prior stroke	0 (0.0%)	0 (0.0%)	-
CHA_2_DS_2_-VASc score	1.3 ± 1.0	1.1 ± 1.1	0.646
Left atrial diameter, mm	36.6 ± 3.0	37.1 ± 3.0	0.612
LVEF, %	64.9 ± 4.1	63.5 ± 4.8	0.373

Values presented are n (%) or mean ± standard deviation as appropriate. AF, atrial fibrillation; BMI, body mass index; LAD, left atrial diameter; LVEF, left ventricular ejection fraction.

**Table 2:** Injection-based occlusion tool performance.

	Number of Occlusion Tool Used	Sensitivity	Specificity	Positive Predictive Value	Negative Predictive Value
Saline Group					
LSPV	31	100.0%	93.8%	93.8%	100.0%
LIPV	36	91.7%	95.8%	91.7%	95.8%
RSPV	29	100.0%	100.0%	100.0%	100.0%
RIPV	42	72.7%	93.5%	80.0%	90.6%
Total	138	92.6%	95.2%	92.6%	95.2%
Glucose Group					
LSPV	31	100.0%	100.0%	100.0%	100.0%
LIPV	37	92.9%	95.7%	92.9%	95.7%
RSPV	27	100.0%	100.0%	100.0%	100.0%
RIPV	40	75.0%	92.9%	81.8%	89.7%
Total	135	93.2%	96.1%	94.8%	94.8%

LSPV, left superior pulmonary vein; LIPV, left inferior pulmonary vein; RSPV, right superior pulmonary vein; RIPV, right inferior pulmonary vein.

## Data Availability

The data used to support the findings of this study are available from the corresponding author upon request.
